# Supplementary figures and images for: Leveraging genetic diversity to identify small molecules that reverse mouse skeletal muscle insulin resistance
Source: eLife. 2023 Jul 26;12:RP86961. doi: 10.7554/eLife.86961 (PMC10371229; doi:10.7554/eLife.86961)

Figure 6-source data

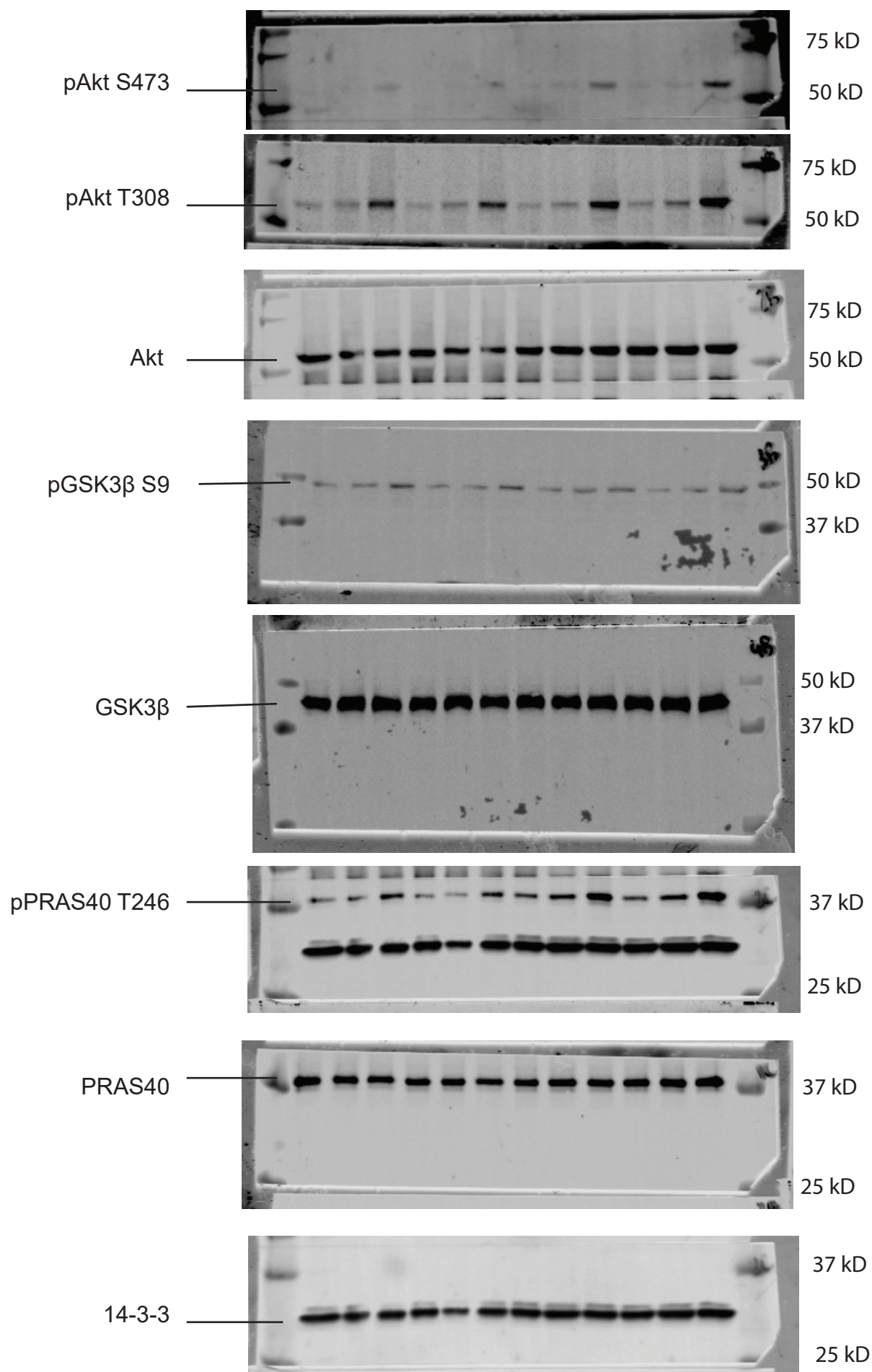

Supplement: Figure 6—source data 1. — Representative immunoblot shown of three independent experiments. [file elife-86961-fig6-data1.zip › Figure 6-source data.pdf]
